# Active multi-point microrheology of cytoskeletal networks

**DOI:** 10.3762/bjnano.7.42

**Published:** 2016-03-24

**Authors:** Tobias Paust, Tobias Neckernuss, Lina Katinka Mertens, Ines Martin, Michael Beil, Paul Walther, Thomas Schimmel, Othmar Marti

**Affiliations:** 1 Institute of Experimental Physics, Ulm University, Albert-Einstein-Allee 11, 89081 Ulm, Germany; 2 Department of Internal Medicine I, Ulm University, Albert-Einstein-Allee 23, 89081 Ulm, Germany; 3 Central Electron Microscopy Facility, Ulm University, Albert-Einstein-Allee 11, 89081 Ulm, Germany; 4 Institute of Applied Physics and Institute of Nanotechnology, Karlsruhe Institute of Technology (KIT), Wolfgang-Gaede-Strasse 1, 76131 Karlsruhe, Germany

**Keywords:** cytoskeleton, intermediate filaments, lock-in technique, microrheology, optical tweezers

## Abstract

Active microrheology is a valuable tool to determine viscoelastic properties of polymer networks. Observing the response of the beads to the excitation of a reference leads to dynamic and morphological information of the material. In this work we present an expansion of the well-known active two-point microrheology. By measuring the response of multiple particles in a viscoelastic medium in response to the excitation of a reference particle, we are able to determine the force propagation in the polymer network. For this purpose a lock-in technique is established that allows for extraction of the periodical motion of embedded beads. To exert a sinusoidal motion onto the reference bead an optical tweezers setup in combination with a microscope is used to investigate the motion of the response beads. From the lock-in data the so called transfer tensor can be calculated, which is a direct measure for the ability of the network to transmit mechanical forces. We also take a closer look at the influence of noise on lock-in measurements and state some simple rules for improving the signal-to-noise ratio.

## Introduction

The dynamic shear modulus describes properties of polymer networks. It can be determined by recording and mathematically transforming the thermal motion of a particle embedded in a viscoelastic medium into the frequency domain. Since no external forces are applied to the motion of the particle, this method is named passive microrheology [1–4]. The resulting shear modulus shows the elastic and diffusive behavior of the investigated medium over the frequency range accessible by the measuring setup. This output is the result of different methods handling the unilateral Laplace transform [5–7]. By exciting a particle with an oscillating force, the shear modulus at a specific frequency can be determined by measuring the response of the particle. The motion of the particle also includes information about the damping and the viscosity of the surrounding medium. This method is known as active microrheology [8–10]. Both the passive and the active method provide an insight into the storage and the loss modulus of the medium. An extension to the single particle active method is achieved by the measurement of two or more particles. It was observed that the movement of two particles embedded in a matrix is correlated [11–12]. There, it was shown for fibronectin that this correlation leads to calculated viscoelastic parameters in good agreement with classical rheology. Actin networks exhibit a similar correlated movement [13]. It was shown that the single-particle and the multi-particle techniques can lead to equal results [14]. For some networks characteristic differences between one-point and two-point microrheology data were found. It was mentioned that inhomogeneities could be determined. An overview over these techniques is given in [15–16].

We present a novel active multi-point microrheology method that allows one to determine the periodic motion of beads in a network responding to an oscillatory excitation of a laser-trapped bead. Our method does not depend on correlations between particle motions, but analyzes the mutual displacements directly. Via the recorded motions of a group of particles located and connected to each other in the viscoelastic medium, it is possible to determine the transfer tensor of motion, the relationship of response amplitudes, phase shifts and frequency changes to higher harmonics with the help of the lock-in technique. A closer look at the signal-to-noise ratio (SNR) is also necessary in order to to ensure applicability of the method over the whole range of experimentally accessible parameters. With this technique we characterized keratin networks with crosslinks of different strength. The concept of the lock-in technique as well as the transfer tensor can be used to improve displacement resolution in all classical single-point and two-point active microrheology methods. However, there will be no contribution to the ongoing discussion about the different results obtained on identical samples by applying different microrheological methods [12,17]. A closer look on the impact of inhomogeneities in networks detected with a similar technique as described here can be found in [18]. Parts of this work were already published in the doctoral thesis of Tobias Paust [19] and in a preliminary communication on the arXiv preprint server [20].

## Results and Discussion

### Theoretical aspects

In this section we have a deeper look at the theory behind the lock-in technique and show how to calculate the transfer tensor. Prior to the determination of the lock-in amplitude one particle has to be trapped and excited by optical tweezers. Then the motion of the excited particle and the particles in the surrounding is captured by a high speed camera and the positions of each particle over time are determined. In a group of particles, one particle – the reference particle *R* – is excited to sinusoidal oscillations at a specific frequency ω, amplitude *A* and direction θ. Consequently, the motion of the reference particle is a superposition of thermal noise and the sinusoidal oscillation. The motion of the response particles is also a superposition of thermal noise, the same sinusoidal function and additionally several terms of the sinusoidal function with doubled, tripled or *n*-fold frequencies. These are the higher order harmonics whose amplitudes depend on the nonlinearity of the system [21] and can be calculated using a Taylor series expansion combined with the addition theorems for sines and cosines. The motion of the response particle can be written as

[1]
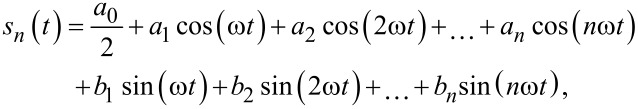


with *a*_0_ to *a_n_* and *b*_0_ to *b_n_* being the Fourier coefficients for the individual terms depending on the *k*-fold frequency. The multiplication of the excitation function *f*(*t*) with sine and cosine, and its time averaging leads to the Fourier coefficients

[2]
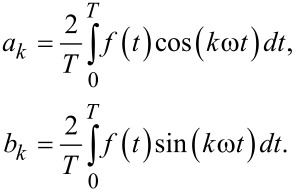


The response function can be expressed as a sum of sine functions under the assumption that the series converges in time. With the coefficients *a_k_* and *b_k_* the relationship of the response amplitudes *x_k_* and phases φ*_k_* to the reference can be gained. In all spatial directions the response vector is

[3]
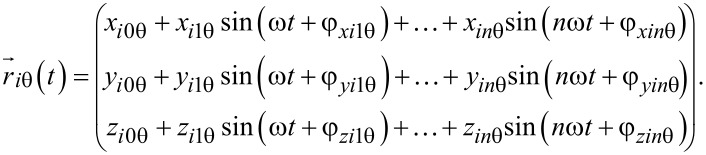


In this equation *x_ik_*_θ_, *y_ik_*_θ_ and *z_ik_*_θ_ denote the amplitudes of the response particle *i* for the specific spatial direction *x*, *y* or *z* with the excitation direction θ at harmonic *k*. The phase information for a corresponding *k* is φ*_xik_*_θ_, φ*_yik_*_θ_ and φ*_zik_*_θ_. The transfer tensor 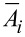, which contains information about the material, links both the reference motion and the response motion. Then it is


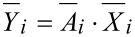


with

[4]
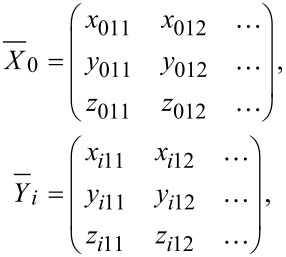


with 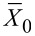 being the reference matrix and 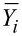 the response matrix of particle *i*. The number of columns of both matrices depends on the amount of excited directions. In order to solve the overdetermined linear equation, the minimum least squares method is taken into account. To infer a unique solution, Equation 4 is transposed and left-hand sided multiplied by the reference matrix. If the reference matrix is left-invertible, the matrix product can be inverted and put on the other side of the equation [22]. This leads to

[5]
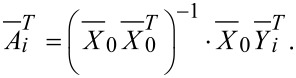


The transfer tensor describes the change of the excitation vector to the response vector of the particles.

### Simulations

**Figure 1:**
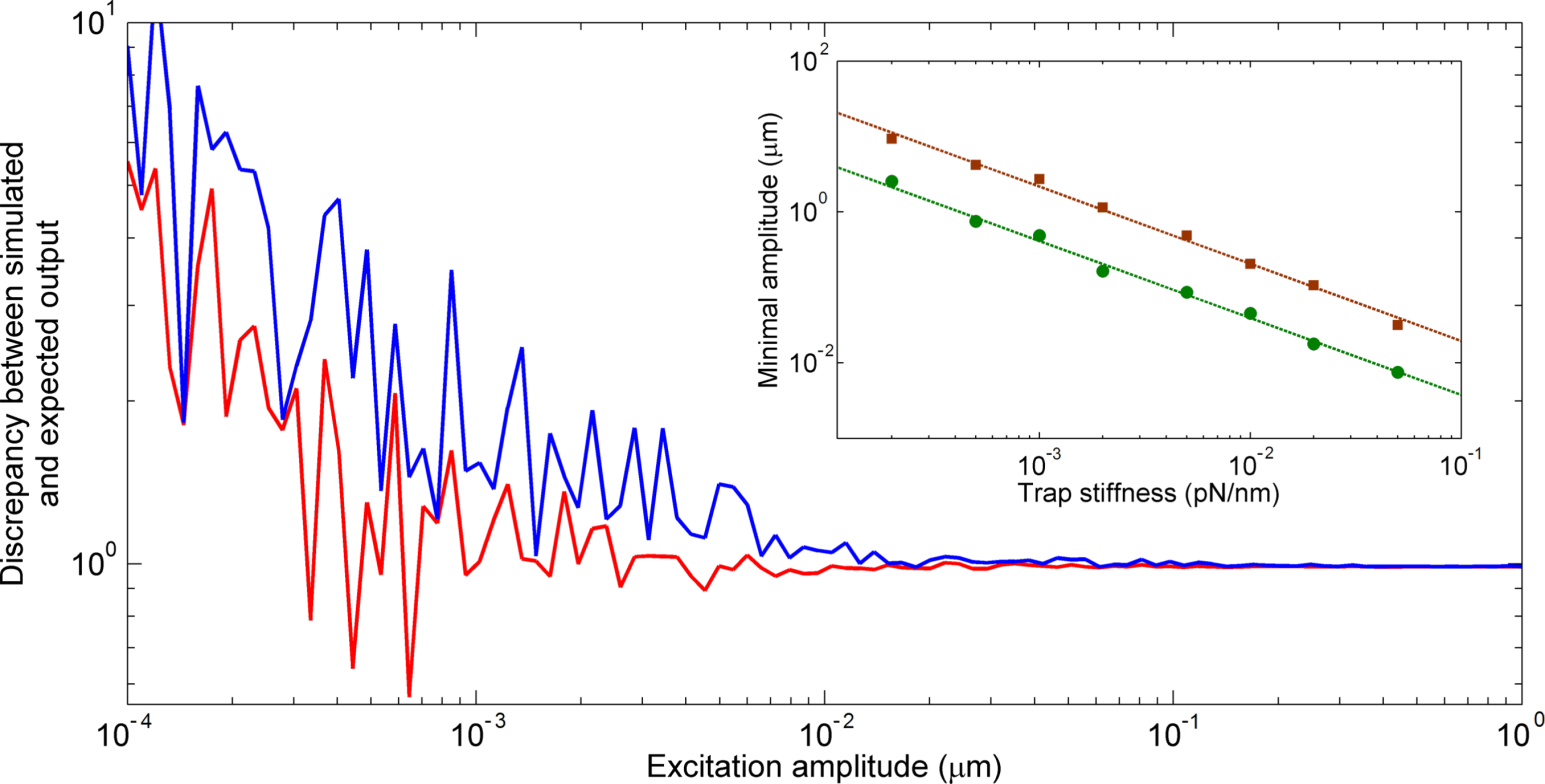
Simulations of the accuracy of the lock-in method. The red line shows the ratio between expected oscillation amplitude and simulated amplitude in the presence of 1% noise. In the case when the expected and simulated amplitudes match, the ratio converges to one. Other values show a misleading amplitude due to a SNR that is too high. With the used parameters the ratio increases for oscillation amplitudes smaller than 10^−2^ μm. The blue line depicts the 5% deviation with 3σ accuracy. Inset: Minimal detectable amplitude, when the calculated amplitude is in the range of 1% (brown) and 5% (green) compared to the expected one (with 3σ accuracy).

The accuracy of a measurement with a lock-in amplifier depends on the noise [23]. Simulations were performed to test how a bad SNR leads to a failure of the method. For this, noise with Gaussian distribution was generated in such a way that the calculated potential matches the theoretical potential of the trap with a stiffness *k*_Tr_ = 1 pN/µm. Afterwards, the noise was added to a generated sinusoidal motion with a frequency *f* = 10 Hz and a data length of 16000 points. The calculation of the SNR in the lock-in method was repeated several times and averaged. Figure 1 shows the ratio between expected and calculated amplitude plotted versus the excitation amplitude. The inset depicts the minimal amplitude, when the calculated amplitude is in the range of 1% and 5% compared to the expected one. This minimal amplitude is determined with 3σ (95%) accuracy and depends on the stiffness of the trap. However, with this method it is possible to measure with trap stiffnesses and applied displacements that are at the lower limit of what is experimentally possible and to still obtain a satisfying SNR.

At an excitation of 10^−2^ µm the calculated amplitude starts to differ from the expected one. This can be seen in Figure 1 where the ratio becomes larger than one. For smaller amplitudes this discrepancy increases rapidly. This means that the SNR is too low to gain a meaningful result from a measurement. In order to improve the SNR and to shift the error to smaller amplitudes, data sets with a length of 10^6^ data points have to be generated. With this change a minimum amplitude of about 1 nm can be achieved. Another possibility to improve the measurement is the change of the trap stiffness. This can be seen in the inset of Figure 1, where a higher stiffness results in a lower minimum amplitude. To reach the best measurement system, both ideas mentioned above have to be considered. If the number of data points and the trap stiffness are low, the lock-in method fails because of a SNR that is too high.

### Measurements

To confirm the functionality of the setup, measurements in bi-distilled water were carried out. The advantage of water is that it is a purely viscous and, even more important, homogeneous liquid, which serves well as a model system for testing the method. In this system a sinusoidal motion of the optical trap leads to a similar motion of the reference particle. A lack of motion of the particle in axial direction confirms that the trap focus stays at constant height. Furthermore, the expected response amplitudes for different angles should always be the same as of the trap. This is valid if the oscillation frequency is small so that the viscosity of the medium does not influence the particle motion.

To show the correctness of the method, reference and response particle are chosen to be the same bead and the transfer matrix of itself is calculated. The calculated transfer matrix is predicted to be the identity matrix because of the calculation with equal matrices. Calculation of the amplitudes corresponding to the higher frequency modes leads to zero because a linear response is assumed. The measured particle motion was in accordance with the calculated particle motion via the lock-in method in the direction of excitation because the Brownian motion was small compared to the oscillations. The diagonal oscillations (45°, 135°) led to amplitudes of 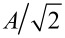. Hence, the measured values were in accordance with theory. The amplitude in the non-excited direction always resulted in minor values. This shows that the method works in the case of a viscous fluid.

Afterwards, the transfer matrix was calculated via the reference and response matrix of the observed motions. This led to


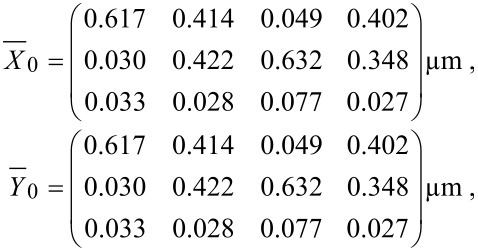


and


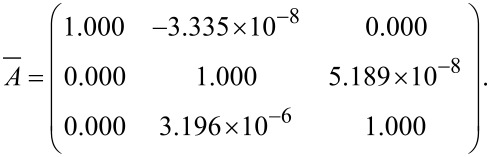


The columns of matrix 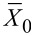 and 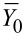 contain the amplitudes (*A*_excited_ = 0.638 µm) at an excitation frequency of 10 Hz for different oscillation angles (0, 45, 90 and 135°). The resulting transfer matrix shows the predicted values apart from rounding artifacts which are in the range of 10^−6^ to 10^−8^.

**Figure 2:**
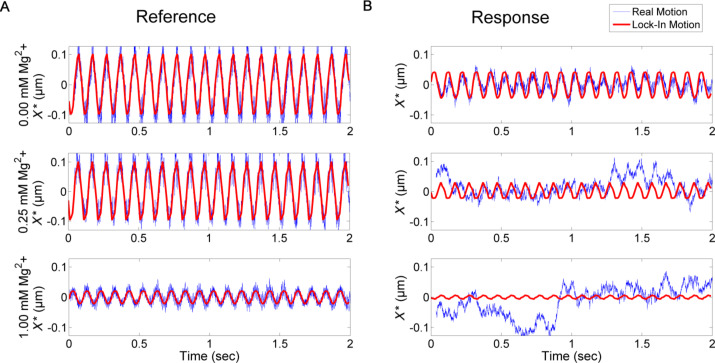
Motion of particles in networks due to the excitation of the oscillating optical trap (*A* = 0.127 µm). The red line denotes the calculated motion via the lock-in method whereas the blue line is the recorded motion. A) Response motion of the reference particle depending on the amount of added crosslinker (laser beam oscillating in the same direction with equal strength). The amplitudes decrease due to a stiffer network. B) Response motion of particles with a distance of 3 µm to the reference particle, dependent on the crosslinker concentration. Since for higher concentrations of Mg^2+^ the reference particle shows a smaller amplitude, the response of a neighboring particle decreases. This implies a decrease of the SNR and introduces errors. The thermal motion becomes more dominant.

Similar to the calculations of the transfer matrix in bi-distilled water, the transfer matrices of three types of in vitro assembled keratin 8/18 networks were determined. The first network was assembled without any additional crosslinker. The second and the third network contained 0.25 mM Mg^2+^ and 1 mM Mg^2+^ as crosslinker, respectively. Since an increasing amount of crosslinker evokes a denser and stiffer network [22], the motions of both the reference and response particles decrease. Furthermore, this results in a change of the transfer tensor and the isotropy of the network. Figure 2 shows the motions of reference and response particle in the direction of excitation for the three different networks.

Without any crosslinker the calculated lock-in motion of the reference particle overlaps with the recorded motion of the CCD camera. A response particle, in a distance of 3 µm to the reference, moves with similar sinusoidal motion as the reference, but with decreased amplitude and phase shift due to the influence of the network. This can be seen for the motion calculated with the lock-in technique as well as for the motion recorded with the camera. In the second case, when a crosslinker is added to the network, the amplitude of the reference motion is smaller than the excitation. While at a concentration of 0.25 mM Mg^2+^ this decrease is very small, at a concentration of 1 mM Mg^2+^ only one tenth of the excitation amplitude deforms the network. At a distance of 3 µm the response motion is reduced because of the decreased excitation and the density of the network. Furthermore, the Brownian motion becomes larger than the response motion, which leads to a decreasing SNR. In the case of 1 mM Mg^2+^ this results in a dramatic increase of calculation errors. The exemplary transfer matrices calculated for particles at a distance of 3 µm for all three cases show that the system is not isotropic. This anisotropy of the assembled network has been investigated thoroughly in [23]. In addition to that, the decrease of the diagonal entries for higher crosslinker concentrations can be explained by lower responses of the particles.


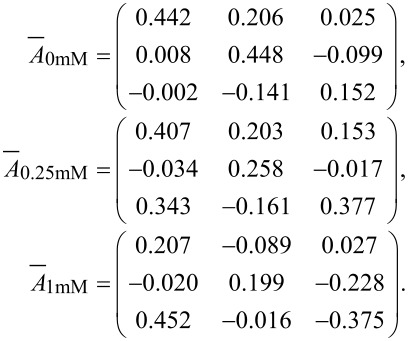


For further calculations the transfer tensors of the networks were separated and averaged dependent on the distance of reference and response particle. Afterwards, a displacement of arbitrary amplitude *a_i_* in the X*-direction, the connecting line between reference and response particle, was chosen to calculate the output vector. The output vector 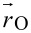 was determined via the multiplication of the arbitrary input vector 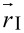 and the average transfer tensor 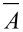 at a specific distance of particles:

[6]
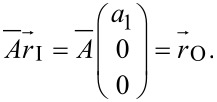


**Figure 3:**
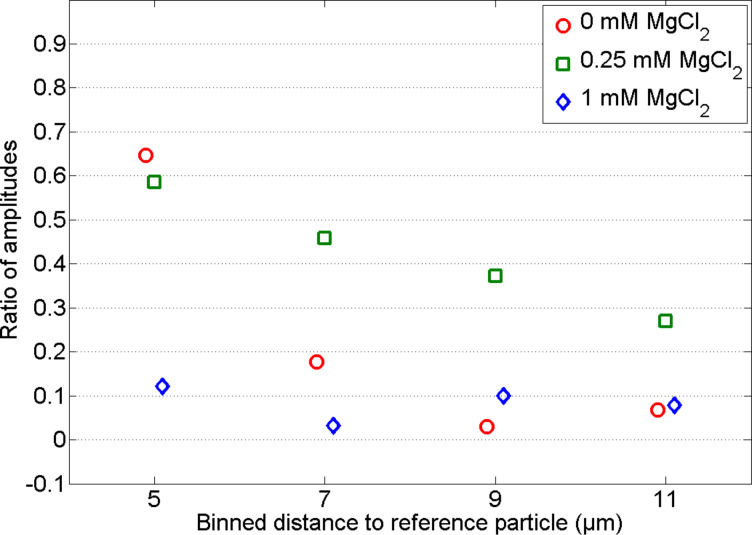
Ratios of excitation and response dependent on the binned distance between reference and response particle. Different markers show varying crosslinker concentrations. Red circles: 0.00 mM MgCl_2_, green squares: 0.25 mM MgCl_2_, blue diamonds: 1.00 mM MgCl_2_. Higher crosslinker concentrations evoke a stiffer network [22].

As ratio the absolute values of output and input vectors 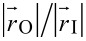 are taken into account and weighted by the standard deviation. Figure 3 depicts the ratio of response and reference amplitude for different distances between response and reference particle. The distance values are averaged over 2 µm bins.

For concentrations of 0 and 0.25 mM MgCl_2_ the ratio of amplitudes decreases, which means that the displacement of the particle gets smaller with increasing distances between reference and response particle. This behavior is quiet intuitive and comparable to theoretical predictions, e.g., from the Thomson model [25]. The larger the distance, the higher the error becomes because the deformation propagation is highly dependent on the morphology of the network. For distances larger than 6 µm networks containing 0.25 mM Mg^2+^ crosslinker show the highest ratios of amplitudes. This means, on one hand, that the network can be deformed more easily than after addition of 1 mM Mg^2+^, and on the other hand, that the SNR is higher so that the errors become less important.

In 0 mM Mg^2+^ measurements the ratio decays very fast to one tenth of the initial value and hence reaches the noise level. This can be explained by a closer look at the network architecture: Without crosslinks the network is very soft and the reference bead can follow the excitation almost completely (Figure 2, low Brownian noise compared to sinusoidal motion). The particles in the direct vicinity of the excited bead also follow the motion almost completely. However, due to the lack of crosslinks the forces are not transmitted well over larger distances.

Measurements at a concentration of 0.25 mM Mg^2+^ show a very smooth behavior, which can be compared to that of the Thomson model [25]. Since this model is for purely elastic materials, one could conclude that for the parameter set chosen (frequency and amplitude of excitation, distance in the low-micrometer range) the network can be well approximated by a continuous elastic material. Although passive microrheology data shows that over large frequency ranges and for small (thermal) forces, the network is viscoelastic [24].

For measurements with 1.0 mM Mg^2+^ the prediction is that the response of the stiff network is very low for short distances, assuming that the deformation force is the same as above. In addition to that the propagation of forces is strongly damped due to the great amount of crosslinks and the very dense network. An additional point is that for such high crosslinker concentrations the diameter of single filaments increases [26] and the network forms thick bundles of different sizes [27], which transmit forces only along their alignment direction. Hence, the assumption of an isotropic medium is not valid and for every position it is not known if the response particle is located in a bundle or next to it. It is also obvious that the errors were very high for the 1 mM Mg^2+^ network. This is due to the low response amplitudes of the reference particle, which deformed the network and lead to a high ratio of reference SNR and response SNR. This resulted in high contributions to the transfer tensor. Between measurements the contributions to the transfer matrices varied a lot and this resulted in the uncertainties.

To improve the results and decrease the error it might be recommended to additionally investigate the amplitudes of the higher harmonics of the measurement. If those amplitudes are comparable to the first harmonic, nonlinear effects play a role, which complicate the interpretations. With the simulation we can calculate the influence of the SNR to the ratio of amplitudes and estimate a lower limit of SNR, below which the error leads to meaningless results. In the case of 1.0 mM Mg^2+^ this limit was exceeded for distances larger than 8 µm so that the response of the particles was not recorded. Only the Brownian motion and surrounding noise was measured.

## Conclusion

With the novel active method new insights into the dynamics of cytoskeletal networks can be gained. Properties such as the response amplitude propagation through the network, the isotropy of the network or the phase change while deforming the network can be determined by one set of measurements. These parameters help in the understanding of network architecture and can be used to estimate the force propagation caused by external stress. In living cells, this force propagation plays an important role for cell migration, reaction to external influences and transport of vesicles. In this work we showed the theoretical idea behind the method, the experimental implementation and in addition its limits via calculations of the SNR. These preliminary results open a wide field of applications in all kinds of different viscoelastic media.

## Experimental

Human keratins 8 and 18 were isolated and purified according to [28–29]. They were assembled into networks in 10 mM Tris-HCl buffer with a protein concentration of 0.5 g/L. Polystyrene beads with 1 µm diameter were incorporated in the network as measuring probes for microrheology techniques and to ensure three-dimensional networks in scanning electron microscopy (SEM). Furthermore, 0, 0.25 or 1 mM MgCl_2_ was added to prepare crosslinked networks with varying stiffness. For further details on network preparation for microrheology see [23]. The equipment used for recording the particle motion is described in [7,24,30].

The laser trapping was realized with an optical tweezers setup consisting of a Nd:YAG laser (Coherent Compass 1064-500) with a wavelength of 1064 nm and maximal power of 500 mW. The used trap stiffness of 9.8 pN/μm was calibrated and adjusted prior to measurement according to [31]. The test of the functionality of the setup was performed in bi-distilled water by observing only the trapped particle.

For SEM measurements the networks were fixed with 2.5% glutaraldehyde (in 0.1 M phosphate buffer with 1% saccharose) for 30 min and contrasted with OsO_4_ (2% in PBS) for 10 min. The filament buffer was replaced by propanol and then the network was critical point dried at 38 °C and 80 bar. The samples were coated with ca. 3.5 nm platinum and imaged with a Hitachi S-5200 scanning electron microscope.

**Figure 4:**
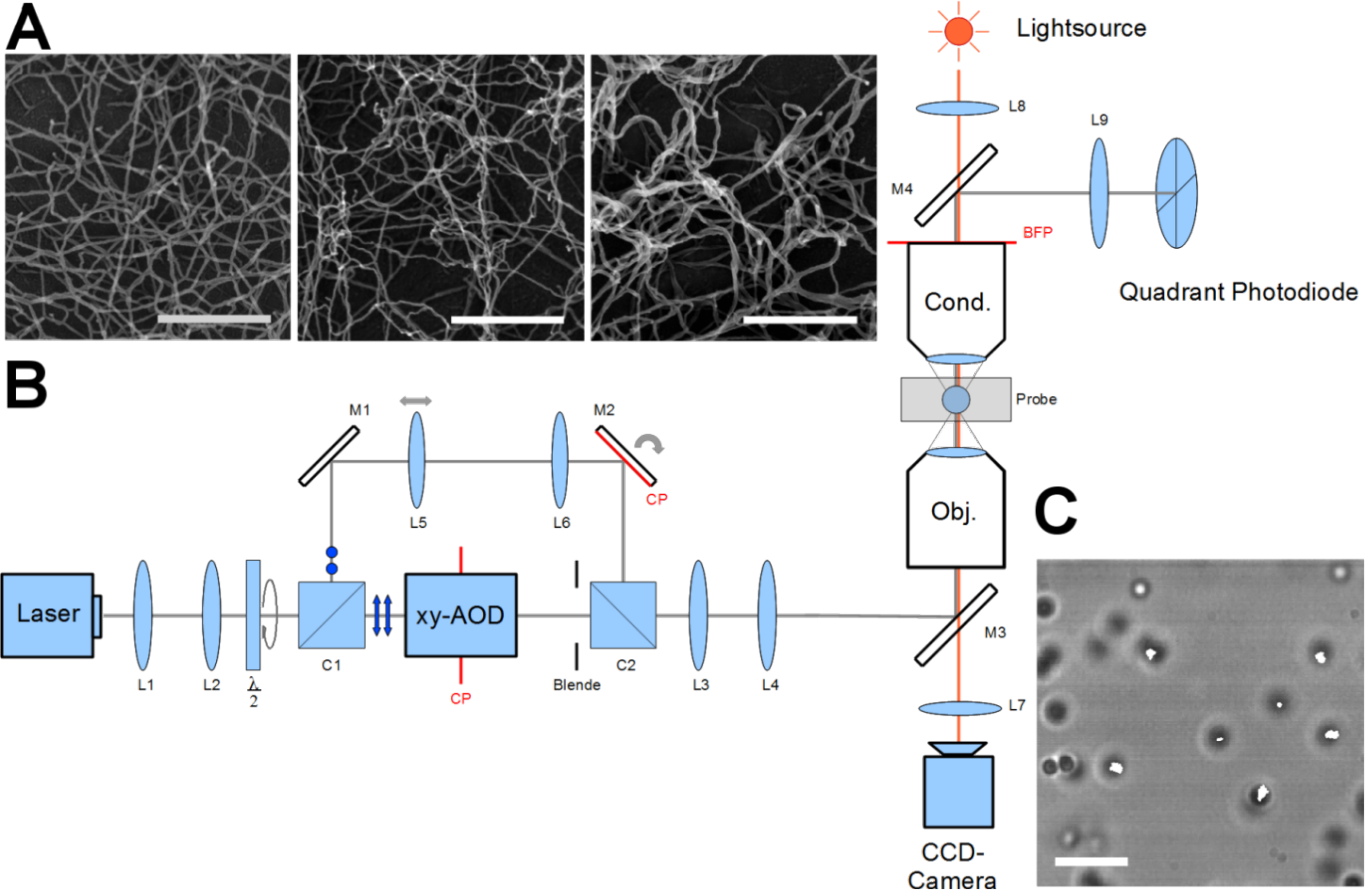
A) Three types of in vitro assembled keratin networks with different amounts of crosslinker (left 0 mM, center 0.25 mM, right 1 mM Mg^2+^). The scale bar for all three pictures shows a length of 500 nm. B) Setup of the measurement device (optical tweezers). The laser beam is focused into the sample to allow for the trapping of microspheres. An acousto-optical deflector (AOD) ensures the oscillation of the laser beam with its pivot in the back focal plane of the objective. The CCD high-speed camera records the motion of the microspheres embedded in the examined medium. An additional photodiode is used for the calibration of the trap. C) Image of a microrheology measurement. The white lines show the trajectories of the particle motion. The length of the scale bar is 10 µm.

For the determination of the transfer tensor at specific media an external force is needed. This external force was applied by using optical tweezers. The excitation of the reference particle to oscillations was realized by the sinusoidal motion of the optical trap. The laser beam is deflected by an acousto-optical deflector to control the movement of the trap. To ensure beam symmetry, the trapping beam pivots at the back focal plane of the objective. The setup of the optical tweezers and a screenshot of the measurement of several particles in an in vitro assembled intermediate filament network are shown in Figure 4B,C. SEM images of the crosslinked networks can be seen in Figure 4A. From left to right the amount of crosslinker increases from 0 to 0.25 and to 1 mM MgCl. With increasing crosslinker concentration the network gets denser. Bundling of the filaments can be also seen for high crosslinker concentrations.
